# Identifying the Validity and Reliability of a Self-Report Motivation Instrument for Health-Promoting Lifestyles Among Emerging Adults

**DOI:** 10.3389/fpsyg.2018.01222

**Published:** 2018-07-19

**Authors:** Min An, Xiao Zhang

**Affiliations:** ^1^School of Education, Linyi University, Linyi, China; ^2^School of Arts and Sciences, University of Rochester, Rochester, NY, United States; ^3^Faculty of Education, The University of Hong Kong, Hong Kong, Hong Kong

**Keywords:** health motivation, health education, health-promoting lifestyles, self-determination theory, emerging adults

## Abstract

**Objective:** Studies on the effectiveness of health-promoting programs across educational contexts need a new tool for measuring health motivation. This study aims to develop a new health motivation questionnaire, namely the College Students’ Health Motivation Questionnaire (CSHM-Q), for college students.

**Design:** An original item pool of the CSHM-Q was developed based on a systematic synthesis and review of related instruments and the content analysis of focus group interviews (*N* = 93). The instrument was then validated using a sample of 205 college students.

**Setting:** Interviews and survey were conducted at three universities in China.

**Methods:** This study explores the content validity, construct validity, and reliability of a self-report motivation instrument based on the framework of Self-Determination Theory.

**Results:** A pilot study showed satisfactory content validity of the motivation constructs and produced 40 items for the CSHM-Q. Results of exploratory factor analysis and parallel analysis further substantiated a three-factor structure of the CSHM-Q. The finalized three-component CSHM-Q has 16 items.

**Conclusion:** Given adequate psychometric properties, the CSHM-Q is a promising measurement tool of health motivation for practical and research purposes.

## Introduction

Lifestyle has a profound impact on people’s health (World Health Organization, 2002). Lifestyle plays a vital role in the prevention of non-communicable diseases (NCDs) such as stroke, obesity, and type II diabetes (World Health Organization, 2006). For example, living in a healthy way at the early stage of life could dramatically reduce the incidence of type II diabetes (Diabetes Prevention Program Research Group, 2002).

Emerging adulthood is a critical life stage for people to develop and maintain a healthy lifestyle throughout life. During this period, individuals gradually form stable habits and lifestyles, which have profound health benefits or threats for their later life (Arnett, 2000). In China and around the world, many emerging adults, typically college students, practice unhealthy lifestyles such as smoking, drinking, sleep deprivation, and sedentary lifestyles (Deliens et al., 2015; General Administration of Sports of China, 2018). These unhealthy lifestyles are risk factors for chronic diseases (World Health Organization, 2005), and may explain up to 75% of chronic conditions (World Health Organization, 2006). Enhancing emerging adults’ motivation to pursue better health plays a key role in encouraging them to adopt healthy lifestyles (O’Donnell, 2012; Seifert et al., 2012). It is difficult to completely explain the complexity of health motivation without inspecting it in various settings and cultural backgrounds. Studies on the effectiveness of health-promoting programs across educational contexts need valid and reliable instruments for measuring health motivation.

In the literature, several measurement scales have been used to measure health motivation. These scales include the Health Self-Determination Index (Cox, 1985), the Health Motivation Assessment Inventory (McEwen, 1993), the General Health Motivation Scale (Thomas et al., 1990), the Motivators and Barriers of Healthy Lifestyle Behavior Scale (Downes, 2008, 2010), the Motivators of and Barriers to Health-Smart Behaviors Inventory (Tucker et al., 2011), the Incentive-Health Promotion Scale (Pascucci, 1992), and the Health Motivation Scale (Xu, 2009). We conducted a systematic review of these scales and found that at least the following issues need to be resolved.

First, most of the existing scales have placed great weight on measuring motivation for physical health behaviors, such as exercise or nutrition behaviors, and ignored other aspects of health motivation. For example, both the Health Motivation Scale (Xu, 2009) and the Motivators of and Barriers to Health-Smart Behaviors Inventory (Tucker et al., 2011) measure only motivation for physical activities and healthy eating. However, health is a multi-dimensional construct that contains physical, mental, and social aspects (World Health Organization, 2009). Scales measuring motivation for multiple aspects of health-promoting behaviors or lifestyles are particularly needed.

Second, most instruments were designed and validated for adult populations. For example, the Incentive-Health Promotion Scale (Pascucci, 1992) was developed for elderly people, and the Motivators and Barriers of Healthy Lifestyle Behavior Scale (Tucker et al., 2011) for African American adults. So far no scales have been designed to measure health motivation among the emerging adult population. Notably, emerging adults (college students in this study) might be a special population for studying motivation for health promoting lifestyles. Compared to their more mature counterparts who are in their 30s or 40s, college students’ healthy choices might be influenced to a larger extent by their parents (Wood et al., 2004), peers (Borsari and Carey, 2001), and public celebrities (Brown et al., 2003). Thus, a measurement tool designed specifically for use in emerging adulthood is needed.

Third, the psychometric properties of the extant measurement scales require further examination (Carter and Kulbok, 2002). For example, the Health Self-Determination Index (Cox, 1985), a frequently adopted instrument by many scholars, was reported to suffer a lack of internal consistency (Carter and Kulbok, 1995; Loeb et al., 2001). Reliability and validity were not reported for the General Health Motivation Scale (Thomas et al., 1990). Furthermore, the length of these instruments might be another issue. Although researchers should choose instruments based on their content rather than their length (Rolstad et al., 2011), evidence shows that a longer test generally causes poorer data quality and lower response rates (Fan and Yan, 2010). Thus, if multiple instruments are available, it makes sense for researchers to choose a shorter one rather than a longer one. This is particular true when researchers use many instruments within one study. In this regard, a shorter instrument is desirable to measure health motivation.

To address each of these issues, in this study we developed and validated the College Students’ Health Motivation Questionnaire (CSHM-Q). We adopted a multi-dimensional perspective of health and emphasized its physically, mentally, and socially integrated nature. To conceptualize health motivation, we considered the basic principles of the Self-Determination Theory (SDT). SDT is a well-known theory of motivation that not only emphasizes goal-fulfillment but also focuses on intrinsically motivated behaviors and satisfaction of basic psychological needs and well-being (Ryan and Deci, 2000). The present study utilized a sample of college students in a collectivist cultural context. College students are navigating the transition to emerging adulthood, which is a critical period for people to make long-term health choices (Nelson et al., 2008). Given that motivation is a key factor in the adoption of health-promoting behaviors (O’Donnell, 2012; Seifert et al., 2012), the health promotion field in higher education will benefit from the development of the CSHM-Q. The validation of this measurement tool will also allow for the in-depth investigation and understanding of health motivation, which will provide a better insight into the implementation of more effective health education programs.

The present study seeks to answer the following research questions: (1) Does the CSHM-Q have satisfactory construct validity? (2) Is this measurement scale reliable for use in Chinese college students?

## Materials and Methods

### Study Design

This study is exploratory in nature and aims to develop a new health motivation questionnaire, the CSHM-Q, for college students and establish its psychometric properties based on the SDT framework. When we designed this study, proactive measures were taken to minimize potential threats to external validity. For example, to avoid sampling error, in which a significant portion of variance in the data is potentially unique to a particular sample, the participants were recruited from multiple universities from south, northeast, and southwest regions of China. The CSHM-Q was developed in a stepwise procedure described in **Table 1**.

**Table 1:** The development of the CSHM-Q.

Steps	Results
Conceptualizing health motivation	• Literature review. • Self Determination Theory; Emerging adulthood; college students. • Five factors: amotivation, external regulation, introjected regulation, identified regulation, integrated regulation.


Generating an original item pool	• Focus group interviews (*n* = 93). • Original items were developed based on a synthesis and a content analysis of group interviews and several relevant instruments. • An item-by-item review by an expert panel, with discussions focusing on ambiguity and relevance; two items were considered irrelevant. • A draft version of the instrument with 40 items was generated.
Preliminary reliability and validity tests	• A total of 24 Items were removed in a stepwise manner based on a series of criteria. • Parallel Analysis in combination with Principal Component Analysis (*n* = 205) suggested a three-factor structure. These three factors were renamed self-focused (8 items), other-focused (5 items), and introjected health motivation (3 items). The finalized scale has 16 items in total. • Reliability test: internal consistency reliability test; test–retest reliability (*n* = 32).

### Setting and Sample

This study included three samples. The first sample included 93 college students (59 males and 34 females, mean age = 21.19 years, standard deviation = 1.36) who participated in focus group interviews. This sample was recruited for the purpose of the content validity analysis. Each focus group included 8 to 10 participants. The second sample included 205 college students (79 males and 126 females, mean age = 19.29 years, standard deviation = 1.33) who participated in a survey. This sample was recruited for the purpose of the factor analysis. Finally, to gauge test–retest reliability, we recruited the third sample which included 32 participants (12 males and 20 females, mean age = 20.78 years, standard deviation = 0.94). All participants were college students at the undergraduate level, single, and recruited from public universities. Participants’ original families lived in urban, suburban or countryside areas. **Table 2** presents details of their demographic information.

**Table 2:** Participants’ demographic information.

	Focus-group interview	Factor analysis	Test–retest study
	Range (Mean, *SD*)	Range (Mean, *SD*)	Range (Mean, *SD*)
**Age**	19–23 (21.19, 1.36)	18–22 (19.29, 1.33)	19–23 (20.78, 0.94)
**Gender**	*Totaln* (%)	*Totaln* (%)	*Totaln* (%)
Male	59 (63.4%)	79 (38.5%)	12 (37.5%)
Female	34 (36.6%)	126 (61.5%)	20 (62.5%)
**Geographic distribution of the participant’s family**			
Urban	30 (32.3%)	83 (40.5%)	6 (18.8%)
Suburban	24 (25.8%)	59 (28.8%)	9 (28.1%)
Countryside	39 (41.9%)	63 (30.7%)	17 (53.1%)
**Academic term**			
1st year	27 (29.0%)	52 (25.4%)	32 (100%)
2nd year	32 (34.4%)	65 (31.7%)	–
3rd year	21 (22.6%)	55 (26.8%)	–
4th year	13 (14.0%)	33 (16.1%)	–
Total	93 (100%)	205 (100%)	32 (100%)

### Ethical Consideration

Before formally conducting the research project, ethical approval was obtained from the Human Research Ethics Committee (HREC) of the Hong Kong Institute of Education (Ref. No. 2012-2013-0210). A team of coordinators who engaged in recruiting and administering the measurement scale received training in ethical considerations. The nature, purpose and ethical issues of the study were explained to the participants and consent forms were obtained.

### Instruments

After reviewing a large pool of related instruments and analyzing the transcripts from focus group interviews, we generated an original item pool for the CSHM-Q.

These instruments we reviewed include the Self-regulation Questionnaire (Ryan and Connell, 1989), Academic Motivation Scale (Vallerand et al., 1992), the Self-Regulation of Withholding Negative Emotions Questionnaire (Kim et al., 2002), the Adolescent Prosocial Behavior Motivation Questionnaire (Wentzel et al., 2007), and the Friendship Motivation Scale (Okada, 2005). Our rationales to review these instruments are as follows. First, all the instruments were developed based on the framework of the SDT. Second, since the current CSHM-Q was designed to measure motivation for practicing health-promoting lifestyles, it is necessary to review the existing instruments that were aimed at measuring motivation for at least one aspect of health-promoting lifestyles. Third, these instruments all target the emerging adult population.

Ten groups of 8–10 college students participated in our focus group interviews. The interviews were audiotaped and transcribed. Open-ended questions (e.g., “Tell me who could influence your choice of a healthy lifestyle?”) that allowed interviewees to talk in any directions were first asked, followed by prompts (e.g., teachers, peers, friends, celebrities, or any other people) in order to probe some interviewees who did not respond. The current study adopted the directed approach (Hsieh and Shannon, 2005) in our content analysis. Before getting started, two student helpers were recruited and trained to get familiar with the operational definitions of motivational levels. In the first coding stage, two student helpers independently coded or identified raw data themes (or items) line by line, and assigned conceptual labels to each of those lines. An inductive analysis was then undertaken to identify more general themes (dimensions or categories) in the second coding phase. Each of these themes (regardless of whether themes were raw data or more general) was considered to be distinct from one another. Disagreements were resolved through further discussion with the first author, and a final consensus was reached.

A panel of seven expert reviewers were invited to review the first draft of the questionnaire. Panel members had various academic backgrounds, including professors in psychology and public health studies, a senior research fellow specializing in educational studies, two Ph.D. students majoring in health education, and lastly, a Ph.D. student majoring in Chinese language studies. Panel members were asked to evaluate the wording and relevance of each item using a 4-point rating scale, and were encouraged to add items that they believed to be important but were absent in the original item pool. The content validity for each item can be determined by evaluating the extent of agreement among different experts (content validity index: i.e., averaging the number of judgments as “relevant” by the total number of panel experts on a particular item; Polit and Beck, 2006). The qualitative findings based on a content analysis of the focus group discussions in this project were published elsewhere (An et al., 2015).

A second draft of the scale was developed based on a synthesis and a review of related instruments aforementioned and the content analysis of group interviews (see Supplementary Table 1).

### Data Collection

Data collection was conducted in several ways: the focus group interviews were implemented through on-site meetings at a public university in south China, and both on-site and online surveys were administered to participants at two public universities in southwest China and in northeast China. Participants were asked to use the CSHM-Q to evaluate their motivation for a healthy lifestyle anonymously. An overview of the study and a consent form were presented before the survey, and participants could choose to finish the paper version of the questionnaire in or after class as per their convenience.

### Data Analysis

Sampling adequacy was evaluated with the Kaiser–Meyer–Olkin (KMO) measure and Bartlett’s Test of Sphericity. Since parallel analysis (PA) is recommended as the most accurate approach for determining the number of components, PA and principal component analysis (PCA) were performed together as a classical implementation (Horn, 1965) to decide the optimal number of components. PA is a resampling technique that treats the original sample as a pseudo-population. Resampling yields a set of average eigenvalues and the 95th percentile eigenvalues from random matrices. Then the original eigenvalues obtained from the data are compared with the eigenvalues obtained from resampling. The 95th percentile of the resampled eigenvalues is equivalent to the significant level of 0.05. The underlying logic is that the factors extracted from the original sample must substantively outperform the factors generated by random chance. PA can be run with exploratory factor analysis (EFA) or PCA, and both approaches have pros and cons. In brief, PA with PCA is prone to under-factor (extracting fewer factors) whereas PA with EFA tends to over-factor (extracting more factors). In other words, the result of PA with EFA may include some meaningless factors (Crawford et al., 2010). We performed the analysis with a viewpoint that a better model is a parsimonious one. In order to avoid over fitting, PA with PCA was chosen for this study.

After the optional number of components was determined, we conduct factor analyses within the FACTOR program using the maximum likelihood method to extract the factors followed by an oblique rotation (e.g., direct oblimin rotation). The Intra-class Correlation Coefficient (ICC) was used to establish test–retest reliability. The Cronbach’s Alpha and McDonald’s ω were adopted to examine internal consistency. FACTOR software was used to conduct PA, factor analysis, and compute ω. Confidence intervals for omega coefficients were computed using the MBESS package within the R (Kelley and Cheng, 2012).

## Results

### Data Cleaning

Once data collection was finished, the authors immediately conducted data cleaning. First, data redundancy was examined, and identical cases or responses were removed and only one row of data was kept in the dataset for analysis. Second, aberrant style pattern was inspected. Data with inconsistent responses, extreme categories, and uniform response vectors were identified and removed. Third, missing value analysis was conducted. The percentage of missing data was 0.24% (less than 5%), and Little’s test (*p* > 0.05) showed the data appeared to be missing completely at random (MCAR); therefore, item means were used to calculate and replace missing values.

### Parallel Analysis

Sampling adequacy was confirmed with the Kaiser–Meyer–Olkin value (0.836). Sufficient correlations for PCA were indicated, as the Bartlett’s Test of Sphericity was highly significant, χ^2^(780) = 4029.79, *p* < 0.001. We first calculated the skewness and kurtosis statistics in order to examine the response distribution of the items: three items showed a non-normal distribution and thus were omitted. We conducted PCA with the varimax rotation method. Nine sub-scales had eigenvalues greater than 1.00, with 64.84% of the variance accounted for. The PA revealed four components greater than chance (**Table 3**), and thus extraction was restricted to four components.

**Table 3:** Parallel analysis in combination with the PCA.

Component	Real-data PCA eigenvalues	PA eigenvalues^∗^	Difference
1	8.37	2.03	6.34
2	4.44	1.87	2.57
3	2.45	1.78	0.67
4	2.01	1.70	0.31
5	1.55	1.62	−0.07

*PCA, principal component analysis; PA, parallel analysis. ^∗^Random data eigenvalues for 100 replications over 37 items and 204 observations.*

### Exploratory Factor Analysis

We only kept items with factor loading values greater than 0.50 and cross-loading values less than 0.32 (Tabachnick and Fidell, 2001; Costello and Osborne, 2005), resulting in the retention of 16 items. However, Component Four included two items that were not interpretable, and we decided to discard this component (see the discussion section for justifications). Three components with 16 items were in line with the SDT framework; the factor loadings and structure were showed in **Table 4**. The three-component solution explained 57.1% of the variance. Based on the theoretical framework and a close examination of the items in each component, we labeled the components as “Self-Focused Health Motivation,” “Other-Focused Health Motivation,” and “Introjected Health Motivation.”

**Table 4:** Item and scale information from the factor analysis with oblimin rotation of a three-factor solution for College Students’ Health Motivation Questionnaire.

Component	Item	Pattern coefficients			Structure coefficients			*M*	*SD*	*h*^2^
		1	2	3	1	2	3			
Self-focused component (1)	11. I enjoy the process of practicing health-promoting lifestyles	0.572			0.605			3.756	1.061	0.403
	14. I practice health-promoting lifestyles in order to keep up a good performance on my study	0.533			0.541			3.654	1.086	0.305
	17. I practice health-promoting lifestyles to affect other people positively	0.739			0.756			3.444	1.082	0.584
	18. Practicing health-promoting lifestyles is another form of filial piety to my parents	0.552			0.571			3.424	0.990	0.360
	23. I practice health-promoting lifestyles because I don’t want to get sick	0.701			0.701			3.951	0.922	0.503
	24. I practice health-promoting lifestyles because I believe health is another form of beauty	0.773			0.766			3.951	1.145	0.592
	34. I practice health-promoting lifestyles because I believe there are strong connections between health and lifestyle	0.759			0.742			3.907	1.027	0.560
	35. I feel pleasure and satisfaction from practicing health-promoting lifestyles	0.801			0.798			3.829	1.017	0.637
Other-focused component (2)										
	06. My teachers told me I should have health-promoting lifestyles		0.526			0.629		2.054	0.935	0.336
	13. I practice health-promoting lifestyles because of the influence from people in public life		0.534			0.577		2.078	0.871	0.433
	20. My parents urge me to practice health-promoting lifestyles		0.790			0.771		2.376	0.934	0.600
	21. I practice health-promoting lifestyles because I had health problems in the past		0.592			0.599		2.463	1.087	0.362
	32. I practice health-promoting lifestyles because other people I am familiar with have health problems in the past		0.617			0.623		2.317	1.044	0.394
Introjected component (3)	22. I feel regretful when I don’t practice health-promoting lifestyles			0.776			0.775	2.502	0.872	0.602
	38. I despise myself if I fail to practice health-promoting lifestyles			0.666			0.641	2.590	0.969	0.423
	33. I feel guilty when I don’t practice health-promoting lifestyles			0.632			0.692	2.654	0.966	0.520
Eigenvalue		4.570	2.937	1.630						
Percentage of variance		28.56	18.36	10.19						
Cronbach α (95% CI)		0.875 (0.847–0.899)	0.758 (0.705–0.805)	0.743 (0.675–0.798)						
McDonald’s Omega (95% CI)		0.878 (0.839–0.916)	0.762 (0.703–0.820)	0.746 (0.673–0.814)						

*h^2^, communalities.*

### Internal Consistency

Internal consistency for all three components and the whole instrument was acceptable. As shown in **Table 4**, McDonald’s omegas were 0.88 for the self-focused component, 0.76 for the other-focused component, and 0.75 for the introjected component. The global CSHM-Q omega was 0.76 (95% CI: 0.68–0.86). Cronbach αs ranged from 0.74 to 0.88, suggesting acceptable internal consistency reliabilities across the three components. In addition, in order to test the homogeneity of the items within each factor, inter-item correlations (0.25–0.48) and item-total correlations (0.72–0.82) were calculated and found to be acceptable. These findings, in combination with results from the exploratory factor analysis, empirically showed a clear internal three-factor structure for the CSHM-Q.

### Test–Retest Reliability

In this study, 32 college students were invited to respond to the CSHM-Q at two time points, with an interval of 3 weeks. The subscale scores of the baseline testing (T1) and the testing performed 3 weeks later (T2) for the CSHM-Q were displayed in **Table 5**. The change and ICC values between these scores were also reported. As shown in **Table 5**, mean differences in subscales over a 3-week interval were 0.22, 0.66, and 0.25, and the *p*-values were 0.71, 0.18, and 0.19 for the self-focused, other-focused, and introjected components of the CSHM-Q, respectively. Thus, there was no significant mean difference in the three components of the CSHM-Q between T1 and T2. Finally, the ICC values were 0.88, 0.79, and 0.87 for the three components, respectively, indicating good test–retest reliability for each subscale of the CSHM-Q.

**Table 5:** Mean scores, across-time mean-level difference, and ICCs.

CSHM-Q	T1 mean (*SD, SE*)	T2 mean (*SD, SE*)	Mean change (*SD, SE*, *p*-value)	ICC	95% CI
Self-focused HM component	20.78 (4.98, 0.88)	21.00 (4.96, 0.88)	0.22 (3.28, 0.58, 0.71)	0.88	(0.75–0.94)
Other-focused HM component	6.38 (3.50, 0.62)	7.03 (2.99, 0.53)	0.66 (2.70, 0.48, 0.18)	0.79	(0.58–0.90)
Introjected HM component	5.63 (1.56, 0.28)	5.88 (1.56, 0.27)	0.25 (1.05, 0.19, 0.19)	0.87	(0.74–0.94)

*T1, baseline testing in the first time point; T2, testing in the second time point (i.e., 3 weeks after T1); P-value, p- value of the paired-samples t-test. ICC, intraclass correlation coefficient; CI, confidence interval.*

## Discussion

The present study showed the plausibility of adopting the SDT for measuring motivation for health-promoting behaviors in Chinese college students. The development of the CSHM-Q makes a significant contribution to health-promoting research and practices in higher education contexts. In addition, the availability of this self-report instrument allows for examining the effectiveness of health education programs on motivation aspects in particular, since motivation is considered a key factor in health-related behaviors (O’Donnell, 2012; Seifert et al., 2012). Information gained from this measurement scale could aid college teachers in improving the efficacy of health-promoting strategies.

Out of the 40 items in the original item pool, 24 were discarded. Justifications of the item reduction are described as follows. First, data normality is a prerequisite assumption for conducting exploratory factor analysis. Thus, an assessment of univariate normality was conducted by examining the kurtosis and skewness of each item prior to exploratory factor analysis. Three items (Item 01, Item 04, and Item 09) were found to exhibit high skewness and kurtosis that exceeded the benchmark range (George and Mallery, 2010). The frequency distribution of Item 01, for example, revealed 174 out of 205 (84.88%) endorsements on Category one and Category two. Item 04 and Item 09 behaved in a similar manner. Second, based on findings from the PA, we ran factor analysis again by imposing a four-factor solution. Items with factor-loadings less than 0.50 and cross-loadings greater than 0.32 were omitted. These items included Items 02, 03, 05, 07, 08, 10, 12, 15, 16, 19, 25, 26, 27, 28, 29, 30, 31, 36, 37, 39, and 40. Ambiguous wordings might be the reason why these items were too difficult to understand for our respondents in this study. Third, Item 07 and item 36 loaded on an independent component that were difficult to interpret based on their literal meanings. Based on a consideration that a component should include at least three items, we decided to impose a three-component solution (Costello and Osborne, 2005). Item 07 and item 36 loaded weakly on any of the components, thus were removed. These procedures resulted in a finalized version of the CSHM-Q with 3 factors and 16 items in total. Results of the finalized CSHM-Q are depicted in **Table 4**.

Based on the findings and the relevant literature, three domains were finally identified and were re-named and re-conceptualized as follows: Domain 1, *Self-focused Health Motivation*, the most integrated form of regulation in which pleasure, happiness, satisfaction, or personal interests are identified as the most autonomous evidence of practicing health-promoting lifestyles; Domain 2, *Other-focused Health Motivation*, indicating that health-promoting lifestyles are externally regulated and are undertaken because of external pressure or the intention to avoid social exclusion because of poor health; The third domain was named *Introjected Health Motivation*, which reflects internal struggle during the internalization and integration process from non-self-determined health-promoting practices to fully self-determined health-promoting behaviors.

It is interesting to point out that items reflecting reasons originated from “others” are referred to as extrinsic motivation (Sheldon and Elliot, 1999) and are expected to load on the other-focused component; however, item 17 (“I practice health-promoting lifestyles in order to affect other people positively”) and item 18 (“Practicing health-promoting lifestyles is another form of filial piety to my parents”) were observed to load on the self-focused component. A possible explanation may be the culture variance on the concept of “autonomous self” between Neo-Confucian collectivism and Western individualism (Lam, 1997). Along with other SDT studies (Kim et al., 2002; Jang et al., 2009), the present findings provide further evidence that, in a Confucian culture, one always has a sense of connection to others, and pursuing social interests is integrated into one’s personal goal fulfillment.

Apart from benefiting future research and enriching our understanding of health motivation, the CSHM-Q is also conducive to the evaluation of healthy-lifestyle promotion programs. Based on a self-determination perspective, the CSHM-Q enables a possible link between motivation assessments and health education. Effective intervention programs could be developed based on a close examination of the relation between health motivation and health-promoting lifestyles. This examination will help enrich our understanding and knowledge about how to promote a healthy lifestyle among emerging adults.

A previous study has revealed a positive relation between intrinsic or pro-religious motivation and health-promoting lifestyles (George et al., 2002). However, in this study, only one participant acknowledged that religious beliefs influenced his choice of health-promoting lifestyles. Because only 2.1% of the Chinese college student population hold strong religious beliefs and participate in religious activities (Liu et al., 2013), we decided to exclude religious factors from this study. Subsequent studies could explore religious influences on health motivations.

Additional psychometric tests could be conducted to further evaluate the CSHM-Q. First, concurrent validity, a subtype of criterion-related validity, could be studied by examining the relation between the scores of CSHM-Q and the Health Self-Determination Index (Cox et al., 1987). Second, convergent and discriminant validity could be evaluated by calibrating the CSHM-Q scores against the scores of Exercise Motivation Scale (Wininger, 2007). Third, continued studies could further examine the measurement invariance (MI) of the CSHM-Q across different groups (e.g., gender, family residence, and ethnicity), as MI is an important requirement that should be established before making any valid group comparisons (French and Finch, 2006). Configural invariance (i.e., a similar factorial structure across groups), metric invariance (i.e., a similar factorial structure and similar factor loadings across groups), and scalar invariance (i.e., a similar factorial structure, similar factor loadings, and similar item intercepts across groups) are three basic types of MI traditionally tested using multi-group confirmatory factor analysis (Van de Schoot et al., 2012; An et al., 2017). Optional new or adjusted approaches such as Bayesian and item response theory methods are also recommended (Van De Schoot et al., 2015). It is also valuable to test the psychometric properties of the CSHM-Q in samples with different cultural backgrounds.

## Conclusion

This study provides substantial evidence for the psychometric properties of the CSHM-Q. The results support a three-factor structure of the scale. We believe that the CSHM-Q is useful in the evaluation of health education practices in higher education and in studies that aim to assess health motivation among young Chinese people. Specifically, the availability of this 16-item measurement scale is helpful to practitioners, such as health education scientists, to better explore different dimensions of health motivation. Further, this instrument can be used by researchers to determine the type of motivation that is significant in predicting the adoption of health-promoting lifestyles by young people.

## Availability Of Data And Materials

Data and materials are available upon reasonable request.

## Author Contributions

MA carried out data analysis, interpretation, and drafted the manuscript. XZ conducted critical revision.

## Conflict of Interest Statement

The authors declare that the research was conducted in the absence of any commercial or financial relationships that could be construed as a potential conflict of interest.
