# Supplementary material for: Identifying the Validity and Reliability of a Self-Report Motivation Instrument for Health-Promoting Lifestyles Among Emerging Adults
Source: Front Psychol. 2018 Jul 19;9:1222. doi: 10.3389/fpsyg.2018.01222 (PMC6060275; doi:10.3389/fpsyg.2018.01222)
Supplement: Supplementary file 1 [file Table_1.docx]

**Original Item Pool Generated from Content Analysis on Group Interviews**

| **No**. | Items |
| --- | --- |
| 1 | I never think of practicing health-promoting lifestyles |
| 2 | I practice health-promoting lifestyles because of the influence from my friends |
| 3 | I practice health-promoting lifestyles because I want to project a healthy image to the others |
| 4 | I practice health-promoting lifestyles I want to improve the quality of my life |
| 5 | The process of practicing health-promoting lifestyles is fun |
| 6 | My teachers told me I should have health-promoting lifestyles |
| 7 | I do not practice health-promoting lifestyles because I just want to enjoy the pleasures of life at present |
| 8 | The facilities and environment that my school provides make me want to practice health-promoting lifestyles |
| 9 | I practice health-promoting lifestyles because I understand the importance of health to my life |
| 10 | I get upset if I don't practice health-promoting lifestyles |
| 11 | I enjoy the process of practicing health-promoting lifestyles |
| 12 | I don’t think of health-promoting lifestyles unless I have health problems |
| 13 | I practice health-promoting lifestyles because of the influence from people in public life |
| 14 | I practice health-promoting lifestyles in order to keep up a good performance on my study |
| 15 | I practice health-promoting lifestyles because I consider it as a way of pressure relief |
| 16 | I feel like a failure of self-control when I don't practice health-promoting lifestyles |
| 17 | I practice health-promoting lifestyles to affect other people positively |
| 18 | Practicing health-promoting lifestyles is another form of filial piety to my parents |
| 19 | It doesn't matter whether my lifestyle is healthy or not |
| 20 | My parents urge me to practice health-promoting lifestyles |
| 21 | I practice health-promoting lifestyles because I had health problems in the past |
| 22 | I feel regretful when I don't practice health-promoting lifestyles |
| 23 | I practice health-promoting lifestyles because I don’t want to get sick |
| 24 | I practice health-promoting lifestyles because I believe healthy lifestyle is another form of beauty |
| 25 | There are other more important things to do rather than health-promoting lifestyles in my life |
| 26 | I practice health-promoting lifestyles because of the influence from my partner |
| 27 | I practice health-promoting lifestyles because I am afraid I cannot afford medical cost for a disease |
| 28 | I think I have to follow the lifestyle tips for my health |
| 29 | I practice health-promoting lifestyles because I want to stay in shape |
| 30 | I don’t practice health-promoting lifestyles because fate decrees life and death |
| 31 | I practice health-promoting lifestyles so other people would be happy |
| 32 | I practice health-promoting lifestyles because someone of whom I am familiar with had health problems in the past |
| 33 | I feel guilty when I don't practice health-promoting lifestyles |
| 34 | I practice health-promoting lifestyles because I believe there are strong connections between health and lifestyle |
| 35 | I get pleasure and satisfaction from practicing health-promoting lifestyles |
| 36 | I don’t think of practicing health-promoting lifestyles because I don't like regular, unchangeable behaviors in my life |
| 37 | I practice it because media influenced my choice |
| 38 | I despise myself if I failed to practice health-promoting lifestyles |
| 39 | Practicing health-promoting lifestyles is one of my habits |
| 40 | Practicing health-promoting lifestyles is a philosophy of life |
